# Multisystemic Granulomatosis Induced by Red Tattoo Pigment: Course Over 10 Years

**DOI:** 10.7759/cureus.107405

**Published:** 2026-04-20

**Authors:** Pascal Cathebras, Cyril Habougit, Sylvain Grange, Mickael Catinon, Michel Vincent

**Affiliations:** 1 Internal Medicine, Saint-Étienne University Hospital, Saint-Étienne, FRA; 2 Pathology, Saint-Étienne University Hospital, Saint-Étienne, FRA; 3 Radiology, Saint-Étienne University Hospital, Saint-Étienne, FRA; 4 Mineralopathology, Minapath Groupe Adène, Lyon, FRA

**Keywords:** atypical presentation of sarcoidosis, multiorgan sarcoidosis, sarcoidosis-like reaction (slr), tattoo complication, tattoo ink reaction

## Abstract

There is a wide spectrum of complications related to tattooing, among them local and regional granulomatous reactions. We report the well-documented case of a a 46-year old male patient who displayed an acute pseudolymphomatous reaction to the red pigment of a tattoo, followed by a systemic granulomatosis involving the skin, lymph nodes, kidneys, meninges, heart, and ultimately lungs and pleura with a refractory, although indolent, course over more than 10 years. The chronological and spatial sequences of clinical and pathological findings, including mineralogic studies, support the hypothesis of a multi-organ granulomatosis induced by an unknown antigen displayed in the red tattoo ink, in an individual genetically and immunologically predisposed to granulomatous inflammation, rather than a more trivial sarcoidosis with tattoo involvement.

## Introduction

There is a wide spectrum of adverse reactions related to tattooing, ranging from mild allergic reactions to serious systemic diseases [1]. Benign local reactions such as itching and swelling, at times sun-related, are common. Non-allergic cutaneous complications of tattoos comprise infections (bacterial, mycobacterial, viral, and fungal), inflammatory reactions of various types (including foreign-body granulomas, vasculitis, sarcoidosis, and pseudolymphoma), and benign and malignant neoplasms [1-2]. Chronic immune-mediated skin diseases, such as psoriasis, vitiligo, lichen, pyoderma gangrenosum, morphea, and sarcoidosis, have a high risk of localization in tattooed skin (Köbner phenomenon) [3-4]. Although lichenoid and pseudolymphomatous histological patterns have been reported in tattooed skin biopsies [1], non-caseating granulomas are more common. Non-infectious granulomatous reactions include foreign-body reactions and sarcoidosis, which are difficult to distinguish, since no histological pattern allows definitive differential diagnosis between these two conditions [2]. Although the involvement of several colors is evocative of sarcoidosis, restriction to one color (mainly black and red) should not rule out sarcoidosis [2]. Most of the tattoo-related sarcoidosis occur in patients with no past history of sarcoidosis at presentation [5]. About 30% of patients with tattoo-related sarcoidosis do not have extra-cutaneous manifestations at the time of diagnosis, but they can be documented later [2]. Between local granulomatous reactions and systemic sarcoidosis lies the condition labeled tattoo granuloma with uveitis (TAGU), which may represent an incomplete form of sarcoidosis or a genuine immuno-allergic hypersensitivity to the components of the tattoo ink [6-7]. Chronic exposure of the immune system to the tattoo’s ink components might trigger systemic granulomatous disease in predisposed individuals [2].

Here, we report a case of severe multisystem granulomatosis likely induced by a red tattoo pigment. We present clinical and histological data with more than 10 years of follow-up, which support the classification of the case as a granulomatous disease induced by the components of tattoo red ink rather than a case of sarcoidosis revealed by a tattoo reaction. The patient has consented to the disclosure of his medical history, photographs, and imaging studies, and the journal has received a signed consent statement.

## Case presentation

**Figure 1:**
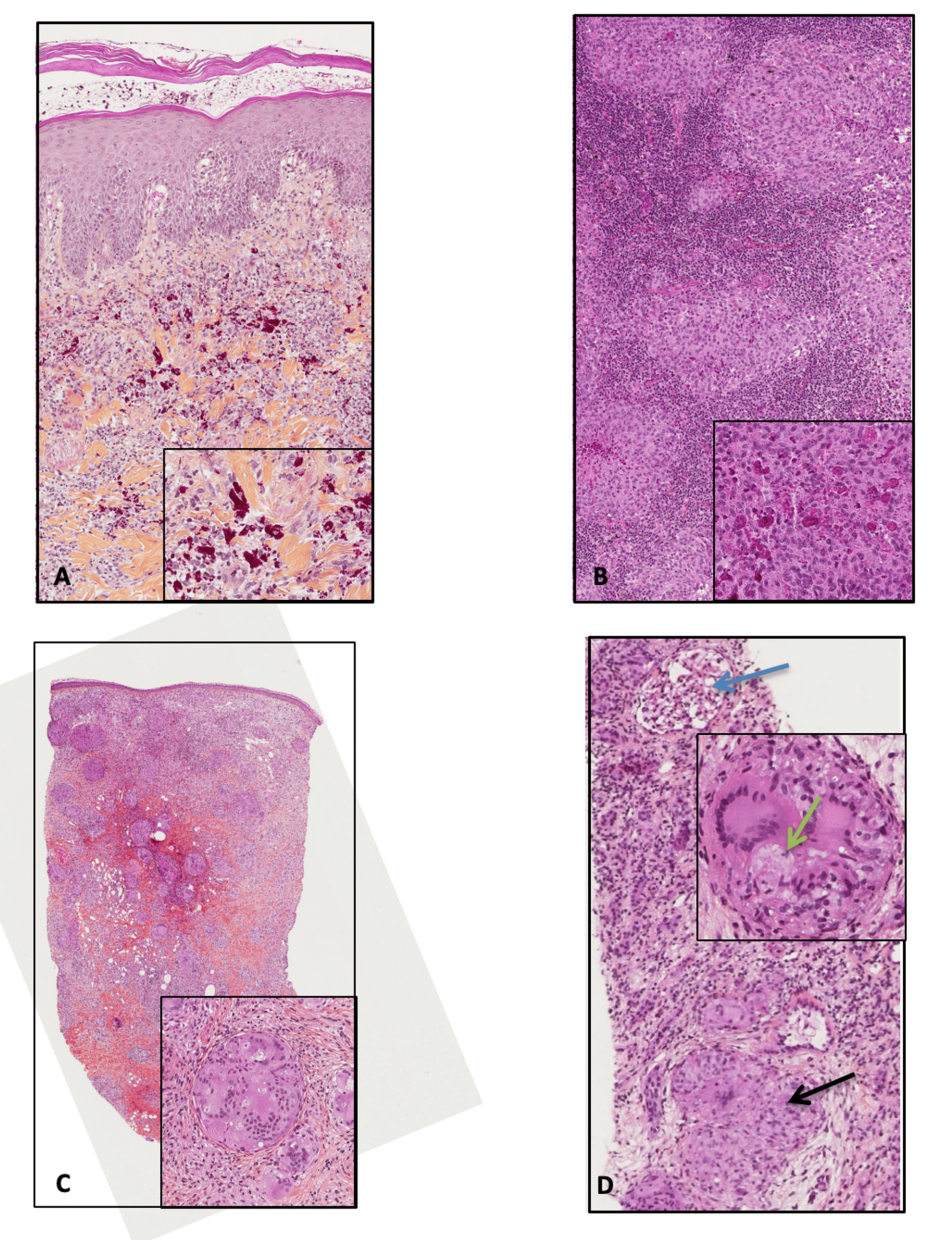
Pathological findings Skin biopsy (2015, left shoulder) (HE ×5) (A) showed a red pigment (inset (HE ×20)) and occasionally pseudolymphomatous reaction in the dermis with regular lymphoid infiltrates (T-cells). Lymph node examination (HE ×10) (B) showed a granulomatosis pattern around red pigment deposits (inset (HE ×20)). Skin biopsy (2019, scalp) (HE ×2.5) (C) identified a granulomatous reaction (inset (HE ×20)) in dermis without pigment deposit. Renal biopsy (HE ×20) (D) identified the granulomatous reaction (black arrow) next to the glomerulus (blue arrow), also without a pigment deposit. An “asteroid” body was seen (green arrow) (inset (HE ×40)).

A man of Caribbean origin, born in 1968, got a tattoo on his left shoulder in 2014. Over the following months, he developed an inflammatory reaction in the red areas of the tattoo and an enlarged homolateral axillary lymph node. On examination, shiny erythematous nodules and plaques were masking the red ink. The first biopsy of the lesion (2015) revealed a red pigment-related pseudolymphomatous reaction in dermis, with lymphoid infiltrates (mainly T-cells) (Figure 1A). The lymph node’s architecture was replaced by granulomas with fibrinoid necrosis, also containing red pigment deposits, which were consistent with a foreign body granulomatous reaction (Figure 1B).

**Figure 2:**
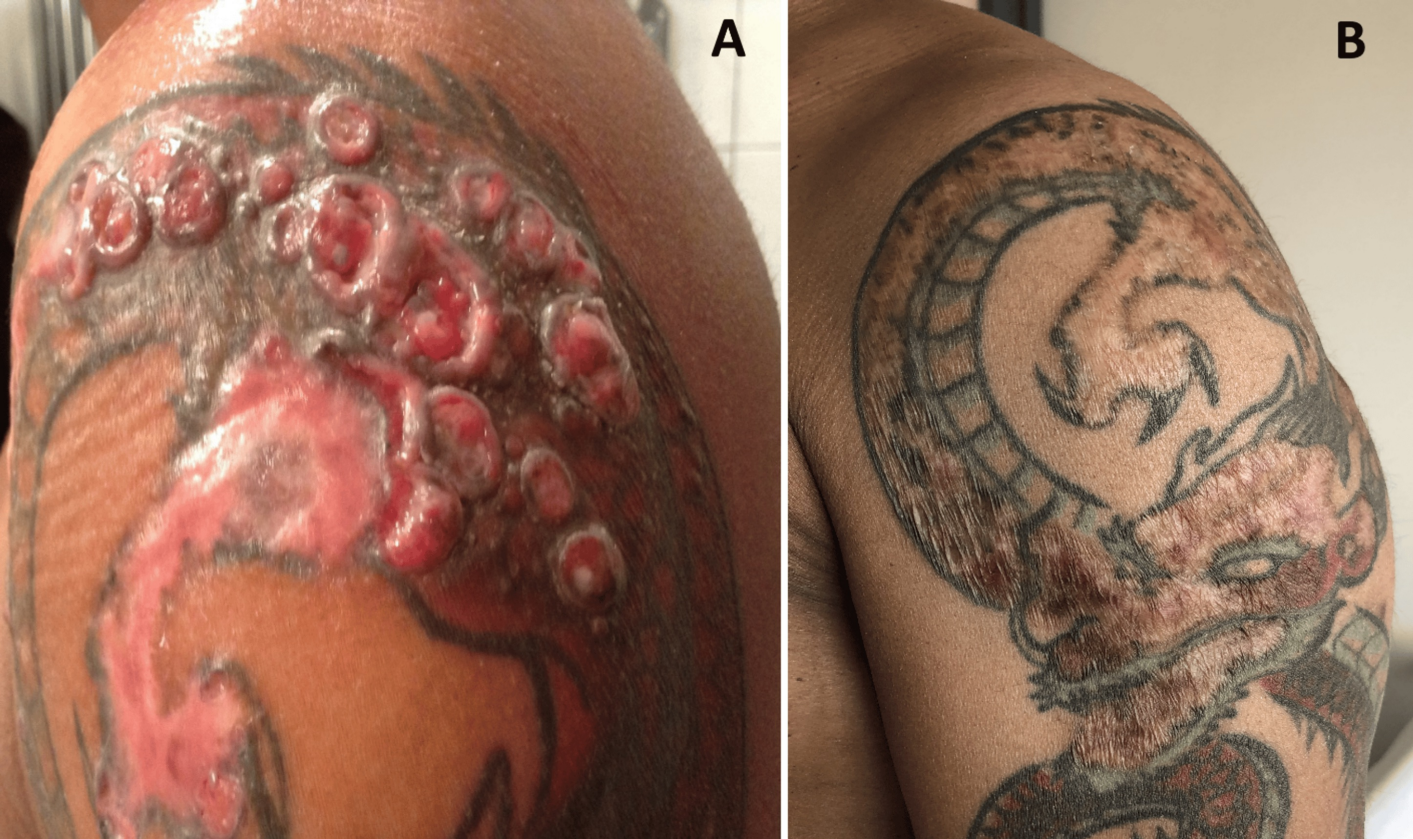
Clinical aspects of the tattoo on the left shoulder (A) Exuberant reaction on the red areas of the tattoo despite treatment with ultrapotent dermocorticoids and a six-month trial of hydroxychloroquine (November 2016). (B) Improvement after seven months of systemic steroid therapy and hydroxychloroquine (January 2020).

Workup for sarcoidosis (lung CT scan, angiotensin converting enzyme) and search for mycobacterial infection were found negative. Despite ultra-potent dermocorticoids (clobetasol propionate for three weeks) and a trial of hydroxychloroquine (400 mg/day for four months followed by 200 mg/day for three months), the cutaneous lesions worsened (Figure 2A).

**Figure 3:**
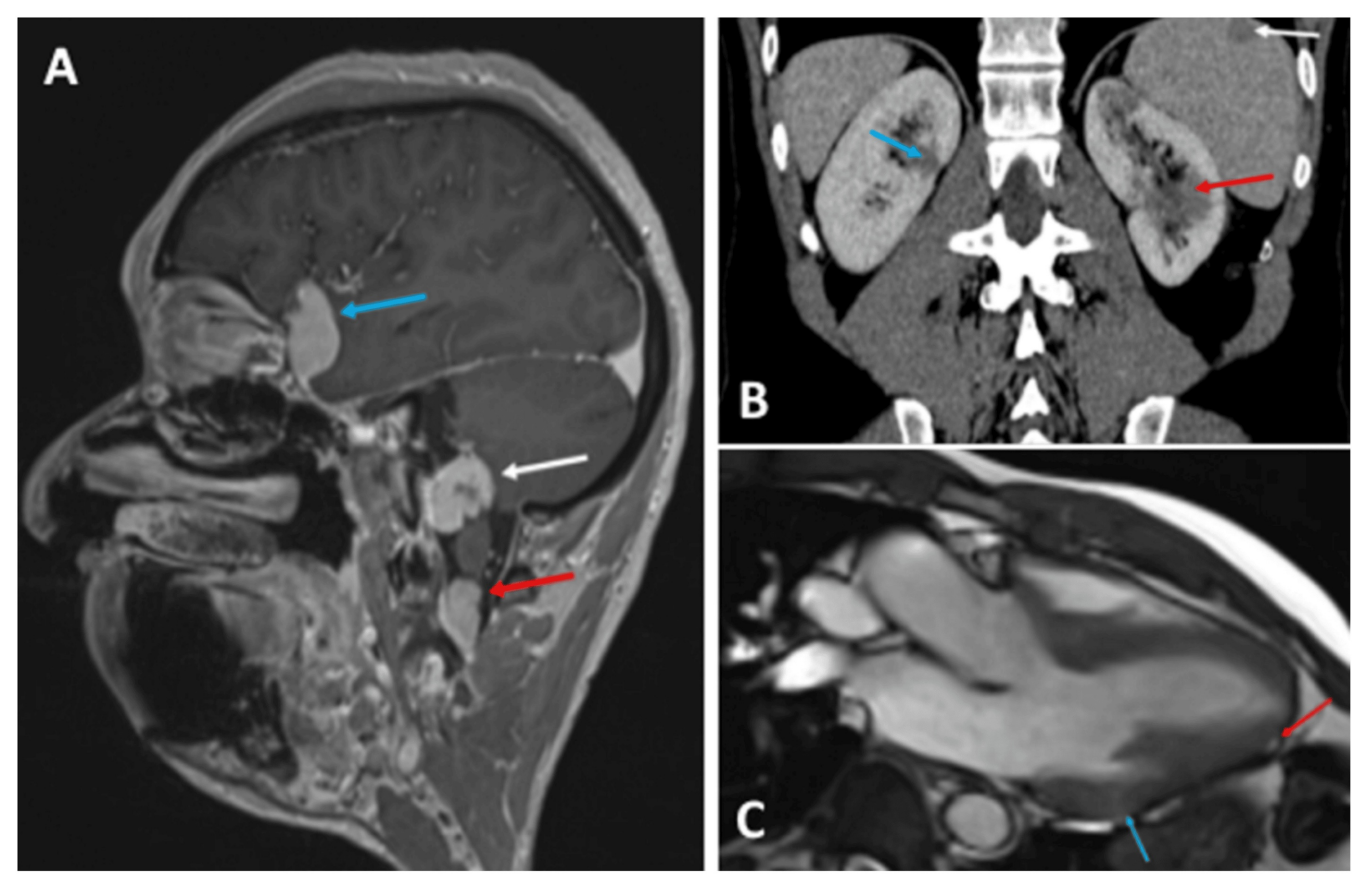
Radiological findings (2019) (A) Brain MRI (enhanced T1sequence) revealing “meningiomatous” cervical (red arrow), infratentorial (white arrow), and supratentorial (blue arrow) lesions. (B) Thoraco-abdomino-pelvic CT scan revealing spleen (white arrow) and kidneys (red and blue arrows) with non-enhanced nodular lesions. (C) Cardiac MRI (T2 sequence) showing endomyocardial infiltrations by focal granulomas next to the apex (red arrow) and the anterolateral papillary muscle (blue arrow).

In 2016, the patient’s EKG showed mild abnormalities (ST-segment elevation in V4-V5); however, cardiac echography and cardiac MRI were normal. In 2018, local injections of triamcinolone (40 mg in February and July) were started. Tender nodules of the scalp were noticed (as the only additional skin lesions), of which a biopsy showed epithelioid and giant-cell granulomas without necrosis or any red pigment (Figure 1C). Brain MRI revealed above and under tentorial multifocal “meningiomatous” lesions (Figure 3A).

Thoraco-abdomino-pelvic CT scan showed a mediastinal lymphadenopathy associated with parenchymatous micronodules, as well as nodular lesions on the spleen and kidneys (Figure 3B). A renal biopsy was in favor of an interstitial granulomatous nephritis (showing epithelioid granulomas, asteroid bodies, and tubular atrophy) without pigment deposit compatible with sarcoidosis. Asteroid bodies were identified in some granuloma (Figure 1D).

Cardiac MRI showed endomyocardial infiltration by focal granulomas in the lateral wall of the left ventricle (here, next to the apex and papillary muscles), highly suggestive of cardiac sarcoidosis (Figure 3C).

All the lesions, cutaneous (Figure 2B) and systemic, partly regressed after systemic steroid therapy (started in June 2019 with prednisone 60 mg/d, progressively tapered as follows: 30 mg/d at three months, 7.5 mg/d at seven months, and 5 mg/d at nine months onward) associated with hydroxychloroquine (400 mg/d). Unfortunately, steroids induced a central serous chorioretinopathy and had to be discontinued earlier than expected. Since the patient remained asymptomatic, no other treatment than hydroxychloroquine was maintained in the long run. During the following years, despite the absence of symptoms, stagnation of imaging was observed at the thoracic, cardiac, renal, and leptomeningeal levels, while a first-degree atrioventricular block and an obstructive pattern on functional respiratory tests were documented. A low-abundance pleural effusion was ultimately discovered in 2024. The workup, including a thoracoscopic surgery, did not reveal an infectious or neoplastic cause, but Hashimoto thyroiditis with subclinical hypothyroidism was found. Pleural biopsies showed nonspecific inflammation without pigment. Under these conditions, adalimumab (40 mg every two weeks) was initiated by the end of 2024, but the chest CT scan did not improve, and by the end of 2025, methotrexate (17.5 mg/week) was introduced. To date, the imaging studies have remained stable.

**Figure 4:**
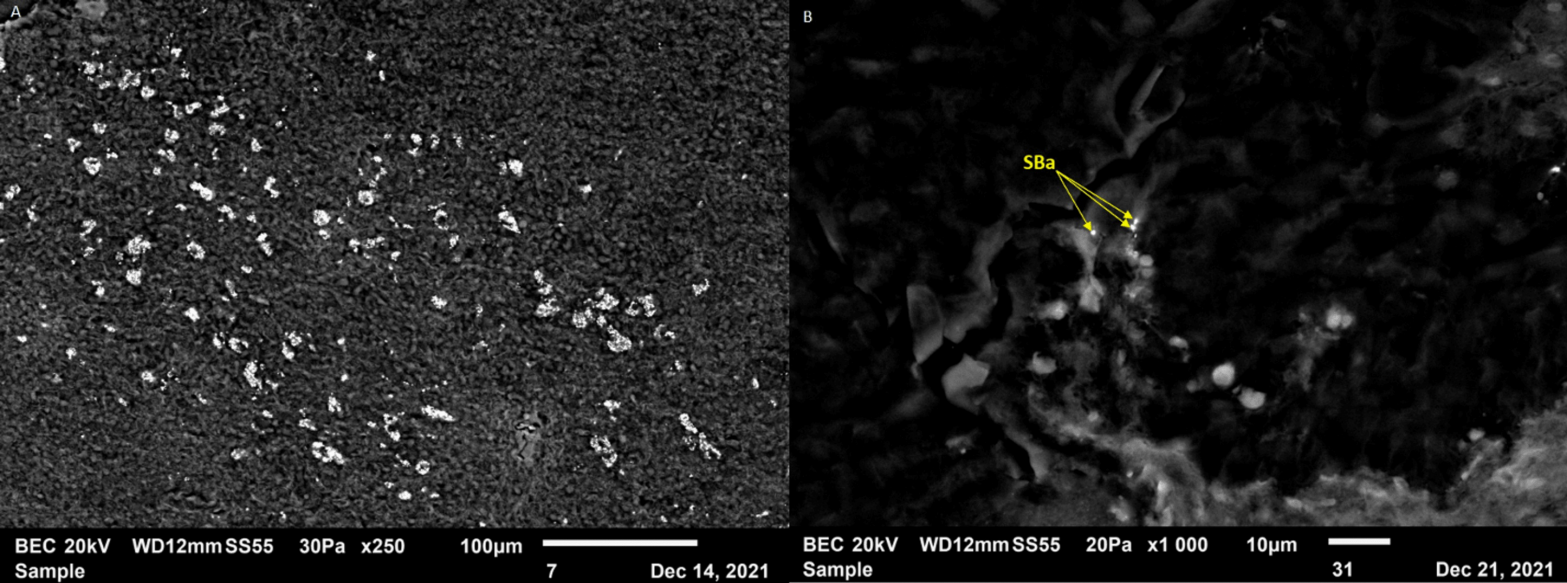
Mineralogic study (A) Observation with a scanning electron microscope of the axillary lymph node biopsy at magnification ×2000 showing a high load of titanium. (B) Observation with a scanning electron microscope of the renal biopsy at ×1000 magnification, showing particles composed mainly of barium sulfate.

In 2021, a mineralogical analysis of the biopsies was carried out with scanning electron microscopy coupled with a spectrometer. At the adenitis level, a major capture of titanium was observed (Figure 4A), while at the renal level, barium particles were highlighted (Figure 4B), these two minerals being usual components of tattoo pigments; the scalp biopsy proved less specific.

## Discussion

Tattoo pigments consist of inorganic colorful metals and their oxides and/or polyaromatic compounds, which are expected to be biologically inert. In vitro chemical analysis of tattoo pigments has shown that the composition of elements in tattoo inks varies greatly, the most commonly identified being aluminium, oxygen, titanium (titanium dioxide is a major component of white pigments), and carbon [8]. Despite the frequent occurrence and wide spectrum of adverse reactions related to tattooing, little is known about the toxicological risks of tattoo pigments, and there are concerns about their potential for phototoxicity, substance migration, and the possible metabolic conversion of tattoo ink ingredients into toxic substances [9]. The risk of cancer associated with tattoos is being investigated at the European level [10]. In addition, it has been shown that metal debris from tattoo needles containing high amounts of chromium and nickel can be transferred into the skin during tattooing [11]. Advanced mass spectrometry methodology has enabled the demonstration of transport of organic pigments, heavy metals, and titanium dioxide from skin to regional lymph nodes [12], which has also been shown in our patient. It is recognized that red tattoo pigments carry a higher risk of adverse reactions [13], although sarcoidosis seems to be mostly associated with black tattoos [14].

In this patient, clinical, radiological, and pathological findings, including some atypical features such as pseudo-meningiomatosis [15], were consistent with sarcoidosis, and the case was managed as such.

Granulomatous infiltration of tattoos by a pre-existing sarcoidosis has been repeatedly reported in the medical literature, although most tattoo-related sarcoidosis occurs in patients with no past history of sarcoidosis at presentation [5]. About 30% of patients with tattoo-related sarcoidosis do not have extra-cutaneous manifestations at the time of diagnosis, but they can be documented later [2]. Such findings raise the hypothesis of “tattoo-induced systemic sarcoidosis” [16].

Whether we should label “sarcoidosis” syndromes characterized by multisystem granulomatous inflammation with various clinical phenotypes and putative etiological factors is a matter of debate between “splitters” and “lumpers” [17]. Considering our case, based on the well-documented history of organ involvement, we believe that the disease should be better understood as a multi-organ granulomatosis induced by an unknown specific antigen displayed in the red tattoo ink, in an individual immunologically and genetically predisposed to granulomatous inflammation. Of particular interest are the chronological and spatial sequences of pathological findings in that case: early pseudolymphomatous reaction to red pigment on the skin, followed by foreign-body type granulomatosis in the regional lymph node with less pigment, and late remote granulomatosis without any pigment (but barium deposits) in the kidneys (and putatively in the heart, spleen, and meninges). No evidence for a systemic sarcoidosis was found at the early stage of the disease (before 2016) despite a complete work-up including chest CT scan and cardiac MRI. The multisystemic involvement was documented later, suggesting a slow disease progression triggered by tattooing. Of course, a causal relationship cannot be inferred from a single case report, and the hypothesis of a systemic sarcoidosis with tattoo involvement cannot be formally ruled out. However, it is noteworthy that the histological findings of the tattoo lesions did not show a granulomatous inflammation, but instead a pseudolymphomatous reaction, which could be interpreted as the first step of the immune process ultimately leading to the multisystemic granulomatosis.

An important limitation of this case study lies in the absence of pathological examination of some involved organs (especially spleen, heart, and meninges), given the risks associated with biopsies. Thus, the granulomatous nature of these lesions remains uncertain, although radiological findings were highly suggestive of sarcoidosis.

Mass spectrometry has demonstrated the transport of organic pigments, heavy metals, and titanium dioxide from tattooed skin to regional lymph nodes [10,12], but the detection of mineral elements known to be part of tattoo pigments at a great distance within granulomatous inflammation (here in the kidney) has not, to our knowledge, been reported until now.

In this patient, the course of the disease over more than 10 years was somewhat refractory, although indolent. The fact that steroid therapy had been shortened because of the occurrence of central serous chorioretinopathy might have contributed to this outcome.

## Conclusions

We have presented a case of multisystemic granulomatosis resembling sarcoidosis, although with some atypical features, initiated by a spectacular reaction to the red pigment of a tattoo. We then believe that the pathophysiology of this condition involves a cell-mediated immune response to an unknown antigen present in the red tattoo pigment. This hypothesis is congruent with the findings of the studies of environmental and occupational risk factors of sarcoidosis, suggesting that the etiologic agents may initiate the disease at very low doses of exposure. The research on potential tattoo-related allergens has shown that the culprit allergens were likely to be formed from a chemical breakdown of the pigment through haptenization, thus the toxicological study of tattoo inks might also fail to identify the specific antigen supposed to have induced the granulomatous reaction.

Among some others, this case report highlights the possible occurrence of multisystem granulomatous reactions with a chronic and treatment-refractory course after the local complication of a tattoo, even with an initial histological pattern of pseudolymphomatous rather than granulomatous reaction. Exuberant reactions to tattoos should prompt investigations to rule out a potentially severe systemic granulomatous disease, whether we should call it sarcoidosis or not.
